# British Society of Gastro-Enterology, Bristol Meeting

**Published:** 1987-11

**Authors:** 


### British Society of Gastroenterology

### Regional spring meeting—Bristol: 19th-20th March 1987

### THE ROLE OF TOTAL PANCREATECTOMY IN THE TREATMENT OF CHRONIC PANCREATITIS

**M. J. Cooper, I. S. Benjamin, A. Cuschieri, I. P. Linehan, R. C. G. Russell, H. B. Torrance and R. C. N. Williamson**
**University Department of Surgery, Bristol Royal Infirmary**

A survey of several British centres collected data on 71 patients undergoing total pancreatectomy (TP) for chronic pancreatitis between 1976–1985. There were 46 males and 25 females with a median age of 38 years (range 19–61 years). Half were alcoholics and half had had previous acute pancreatitis. Besides jaundice (17%) severe pain was the indication for operation; regular opiates were needed in 82% of patients and 36% were addicted. All but 10 had had previous pancreatobiliary surgery, with a median of 2 (maximum 6) operations. TP was a one-stage procedure in 28 patients, 37 having had distal and 6 proximal resections in the past, the pylorus was preserved in 26. Median operative time was 4 hours (range 2–18 hours) and blood loss 3 units (1–21 units). Intraoperative complications in 12 patients included haemorrhage in 9. Four deaths occurred within 30 days from bleeding (2), respiratory failure (1) and Roux-loop infarction (1). All but 1 of the 67 survivors required full pancreatic supplementation and 39% had difficulties in endocrine control. At a median follow-up of 2 years (range 0.25–10 years), 44 patients (62%) were pain-free and 8 (11%) needed only occasional analgesia. Though 15 (22%) still took regular analgesics, all were symptomatically improved. There have been 9 late deaths (13%). TP produces effective analgesia in chronic pancreatitis with an acceptable mortality rate (5.6%), but operative technique and postoperative care are demanding.

### SMOKING DELAYS THE ONSET OF ULCERATIVE COLITIS

**R. J. Motley, J. Rhodes, S. Kay and T. J. Morris**
**University Hospital of Wales, Cardiff**

Two hundred and eighty eight patients with ulcerative colitis completed questionnaires about their smoking habit. Approximately half were male and half female. A third of the males and two-thirds of the females were life long non-smokers before developing colitis. Male non-smokers were younger and developed their colitis much earlier than males who had smoked before the onset of their disease (mean age difference 13.8 years). There was little difference between female smokers and non-smokers. The time at which colitis began correlated with the total number of years patients had smoked. There was a high incidence of new patients during the years immediately following cessation of smoking (87 patients presented within 12 years and 29 of these presented within 2 years). The findings suggest that smoking probably delays the presentation of colitis in males. The site of colitis defined as rectum only, left sided or total, was not related to the patient's previous smoking history.

### GASTRIC EMPTYING AFTER CONSERVATIVE PANCREATECTOMY

**M. J. Cooper, I. Stewart, E. Rhys-Davies, R. C. N. Williamson**
**University Department of Surgery, Bristol Royal Infirmary**

Pylorus-preservation during pancreatoduodenectomy (PPPP) should retain a normally functioning stomach and reduce post-operative side effects. Gastric function was assessed in 21 patients (10M:11F, median age 41 yrs, range 28–81 yrs) who had undergone PPPP since 1981 for chronic pancreatitis (n = 11) or localised neoplasia (n = 10). Reconstruction by end-to-side duodenojejunostomy preserved a median 3 cm of duodenum. There was one postoperative death and 2 patients required early reoperation; the remaining 18 patients returned to a normal diet at a median of 8 days (range 5–25 days). At a median follow-up of 19 months (range 3–48 months) 17 of the 20 survivors (85%) were adjudged to be Visick grades I or II. Bile reflux was demonstrated in 6 of 12 patients by BIDA scan, although it was confined to the antrum in 5. Gastric emptying (GE) of both solid and liquid meals was assessed scintigraphically in 14. In 2 patients gross prolongation of GE (>200 min) led to surgical revision, one for recurrent pancreatic cancer at the anastomosis and the other for gastric atony 15 months after resection for pancreatitis. Six asymptomatic patients had moderate prolongation of GE (>140 minutes for solids) whilst the other 6 were normal (<90 min). No peptic ulcers have been seen. Asymptomatic prolongation of GE is the only apparent adverse effect of PPPP, but loss of the duodenal pacemaker may occasionally necessitate reoperation.

### CARDIFF CROHN'S DISEASE JUBILEE—A PRELIMINARY REPORT

**J. D. R. Rose, G. M. Roberts, G. Williams, J. F. Mayberry and J. Rhodes**
**University Hospital of Wales, Cardiff**

A register of all patients with Crohn's disease resident in the City of Cardiff at the time of diagnosis has been compiled for the period 1932–1985. It contains 408 patients with a male:female ratio of 1:1.38. The terminal ileum was most commonly affected (59%), followed by the left colon (29%), right colon (17%), anus (13%), diffuse small bowel disease (11%), gastroduodenum (1.2%) and other sites rarely (0.7%). New cases increased by decade from 3 in the period 1936–1945, 25 in 1946–1955, 59 in 1956–1965, 126 in 1966–1975 to 187 in 1976–1985. Although terminal ileal disease increased with each decade, its proportion declined steadily from 100% in 1936–1945 to 55% in 1976–1985. Similarly, the number of diagnostic laparotomies increased prior to 1966–1975 but the proportion fell from 100% to 25% between 1976 and 1985. The peak age at diagnosis for both ileal and colonic disease was 20–24 but the proportion of ileal disease fell from 75% at ages 15–19 and 20–24 to 36% in the age groups 75–79. These data demonstrate a continuing increase in Crohn's disease with a trend towards more colonic disease particularly in the elderly.

### IMAGING IN THE PRE-OPERATIVE DIAGNOSIS OF MECKEL'S DIVERTICULUM

**P. M. Dixon and D. J. Nolan**
**Department of Radiology, John Radcliffe Hospital, Oxford**

There have been few comparative reports of the techniques available for the pre-operative diagnosis of Meckel's diverticulum. $^{99m}$Tc pertechnetate scintigraphy is claimed to have a 85–90% sensitivity, but many of these studies were carried out in children and the techniques may be much less reliable in adults.

We reviewed all adults with surgically confirmed Meckel's diverticulum, presenting over a ten year period. The use of nuclear medicine, small bowel enema and angiography in the pre-operative period were assessed.

$^{99m}$Tc pertechnetate scintigraphy detected one case out of six examined. Small bowel enema showed the diverticulum in six patients and confirmed small bowel obstruction in a seventh. One patient had a normal study. Selective angiography was used in cases of active bleeding and was positive in two patients out of three examined.

The small bowel enema appears to be the most accurate technique for detecting Meckel's diverticulum. Nuclear medicine techniques are useful in children but the sensitivity in adults is less than is widely believed. Angiography is useful in cases of profuse bleeding.

### IMMUNOREACTIVE METALLOTHIONEIN IN COPPER-LOADED HUMAN LIVER—A HISTOPATHOLOGICAL STUDY

**M. E. Elmes, N. J. Mahy, B. Jasani, J. P. Clarkson**
**Pathology Department, University of Wales College of Medicine, Cardiff**

A retrospective study of the distribution and staining intensity of immunoreactive metallothionein (MT) was carried out on 91 liver biopsies using a sensitive immunoperoxidase technique and copper and copper-associated protein using rubeanic acid and orcein stains. 66 biopsies were from patients with conditions associated with copper retention; 25 were histologically normal. The results were correlated with the presence of cholestasis, fibrosis and features of hepatocellular damage. Normal livers contained no demonstrable copper or copper-associated protein and a centrilobular distribution of cytoplasmic MT of moderate intensity. Cholestasis correlated with a more diffuse distribution; bile plugs were frequently MT positive. In piecemeal necrosis the degenerate hepatocytes were strongly MT positive; cirrhotic livers showed a paraseptal distribution of MT positive cells and low overall intensity. Copper and copper-associated granules were frequently MT positive but were a small part of the total MT. Strong MT staining of hepatocytes was found in primary biliary cirrhosis but the most intense MT staining was found in hepatocytes in Wilson's disease. This was reduced in one post-treatment biopsy. Immunocytochemical demonstration of MT provides important supplementary information in liver biopsy interpretation in disorders leading to copper retention and may be useful in diagnosing Wilson's disease.

### THE BLOOD TRANSFUSION EFFECT AND COLORECTAL CANCER

**W. B. Ross**
**Derbyshire Royal Infirmary and Cardiff Royal Infirmary**

Perioperative blood transfusion may cause immunosuppression in cancer patients and this could have a detrimental effect on their survival. During a four year period, 314 patients had surgery for colorectal cancer. Complete data sets were retrieved for 302 cases. Of these patients, 25 died within 30 days of surgery, 41 others had unresectable tumour or metastases found at laparotomy and 236 patients had curative resections. The 77 patients who had abdomino-perineal resections were excluded to limit analysis to the 159 patients who had abdominal resections. Perioperative blood transfusions were given to 95 (60%) of these patients. This group was compared with patients who were not transfused. Age, clinical stage, histological grade and tumour size were similar in both groups, but there was a higher incidence of males, left-sided tumours and tumours adherent to other structures in the transfused group. The 5-year survival was 34% in those transfused and 47% in those who were not. The logrank test did not demonstrate a significant difference between the survival curves. In contrast to others, this study cannot support the theory that blood transfusion may have a detrimental effect on survival in colorectal cancer. Further study of this problem is indicated.

### CELL-MEDIATED IMMUNITY IN COELIAC DISEASE: RESPONSE TO A SYNTHETIC DODECAPETIDE SEQUENCE FROM A-GLIADIN

**J. A. Karagiannis, J. D. Priddle and D. P. Jewell**
**Gastroenterology Unit, The Radcliffe Infirmary, Oxford**

A region of A-gliadin from Scout 66 wheat (residues 206–217) resembles part of protein Elb of human adenovirus 12 (residues 384–395). We have shown that a synthetic peptide (A-gliadin 206–217) mediates cellular immune responses in patients with coeliac disease in remission but not in healthy subjects or in the majority of patients with inflammatory bowel disease. We now report on serial observations from 5 patients newly diagnosed as having coeliac disease.

The indirect leucocyte migration assay was used in which peripheral blood mononuclear cells from patients were incubated with the dodecapeptide at concentrations of 33, 11, and 55 μg/ml. The release of migration inhibition factor was assayed by its effect on the migration of leucocytes from healthy subjects under agarose. The five patients had subtotal villous atrophy on the initial biopsy and after 3–5 months of a gluten-free diet showed an excellent histological response. Using 33 μg/ml, MI was 0.96 (SD 0.08) before treatment and 0.79 (SD 0.07) after ($p<0.01$). For 11 μg/ml the values were 1.06 (SD 0.11) and 0.85 (SD 0.10) respectively, ($p<0.02$) and for 5.5 μg/ml, 0.96 (SD 0.06) and 0.91 (SD 0.11) respectively ($p>0.05$). Thus, peripheral blood mononuclear cells show little response to the peptide before treatment, but following a gluten-free diet, the cells show immunological responsiveness in a dose dependent manner. This parallels earlier observations using gluten fractions.

### PANCREATIC ARTERIOGRAPHY: DOES IT HAVE A USEFUL ROLE?

**G. V. N. Appleton, N. Bathurst, J. Virjee, R. C. N. Williamson**
**Departments of Surgery and Radiology, Bristol Royal Infirmary, Bristol**

Selective visceral angiography should help to determine the nature and extent of pancreatic lesions and their suitability for resection. Between 1980–86 coeliac and superior mesenteric angiograms have been obtained by transfemoral cannulation in 71 patients considered for pancreatectomy (mean age 50 years), including 44 with malignancy and 28 with obstructive jaundice. Anomalous arterial anatomy was delineated in 20%, notably an aberrant origin of the right hepatic artery (14%). Among arterial changes observed in 41 patients (59%), increased or decreased vascularity and displacement were of limited diagnostic value, but encasement correctly predicted cancer in 17 of 19 cases and irresectability in 9 of these. When present (17%), invasion or occlusion of the superior mesenteric or portal vein was more sensitive, indicating cancer in 11 of 12 cases and irresectability in 10 of these. Hepatic metastases were only detected in 7 of 15 patients (47%). Overall angiography confirmed the diagnosis in 53%, localised the lesion in 69% (including 3 of 5 insulinomas) and correctly forecast irresectability in 65%; misleading data were obtained in 4 patients. There were no significant complications. Pancreatic angiography is a safe technique which contributes to the assessment of disease and the planning of operation, particularly when it shows arterial variation or venous obstruction.

### ARE DUODENAL DIVERTICULA ASSOCIATED WITH CHOLEDOCHOLITHIASIS?

**R. H. Kennedy and M. H. Thompson**
**Department of Surgery, Southmead Hospital, Westbury on Trym, Bristol**

Morbidity is only occasionally attributed to duodenal diverticula and they are not generally considered to be associated with choledocholithiasis. This assumption was reassessed by the analysis of patients undergoing endoscopic retrograde cholangio-pancreatography (ERCP) during a 3 year period. The results of 245 consecutive duodenoscopies were analysed. Cholangiography revealed common bile duct stones in 70 patients of whom 24 (34%) had periampullary diverticula. Clear bile ducts were shown in 92 cases of whom only 12 had diverticula (13%). This difference is statistically significant ($P<0.01$). In the remaining 83 patients cholangiography was not successful for technical reasons or was not indicated: further clinical follow-up and/or investigation have failed to reveal duct stones in any. Only 11 (13%) of these patients had diverticula. Of the 47 patients who had diverticula, 24 had duct stones, 5 had none but were thought to have recently passed stones, and 18 had no calculi.

These results reveal a significant association between periampullary duodenal diverticula and choledocholithiasis, although they do not shed light on the disease mechanism involved. Diverticula may predispose to the formation of duct stones, or may mechanically impede the passage of stones through the sphincter of Oddi.

### GRANULOMATOUS LIVER DISEASE—AN UNUSUAL CASE, AND A REVIEW OF THE PAST 3 YEARS EXPERIENCE

**A. Williams, M. Hall and B. Cooper**
**Department of Medicine, Bristol Royal Infirmary, Bristol**

A 43 year old white male presented with a three week history of leg and scrotal swelling, jaundice, pale stools, and dark urine. He was deeply jaundiced with an enlarged firm liver. Extrahepatic biliary obstruction was excluded. His CXR was abnormal and an isotope liver scan showed changes consistent with secondaries. A diagnosis of carcinomatosis was made.

However, endobronchial and liver biopsies showed granulomata, Langhans giant cells, and fibrosis. A diagnosis of sarcoidosis was further supported by a negative mantoux, hypercalciuria, elevated serum angiotensin converting enzyme, and a characteristic gallium scan. A kveim test was not performed as treatment with corticosteroids was commenced. The patient subsequently developed a cardiomyopathy with complete heart block, and neurological complications. Acute intrahepatic cholestasis is a unique presentation of sarcoidosis, but chronic cholestasis although rare is well described in Africans. We reviewed all the cases of granulomata on liver biopsy at our hospital in the past 3 years. There were 3 cases of primary biliary cirrhosis, 3 of idiopathic granulomatous hepatitis, 2 of sarcoidosis, 1 of Hodgkins disease, and 1 of tuberculosis in association with acute myeloid leukaemia.

### HEPATIC VENO-OCCLUSIVE DISEASE FOLLOWING COMFREY INGESTION—CASE REPORT

**C. F. M. Weston, D. F. Levine*, J. D. Davies and B. T. Cooper**
**Departments of Medicine and Histopathology, Bristol Royal Infirmary, Bristol and West Cornwall Hospital*, Penzance, Cornwall**

We investigated a 13 year old boy presenting with abdominal swelling and hepatomegaly. He had been found to have Crohn's disease of the terminal ileum and colon 3 years previously and had received conventional treatment with prednisolone and sulphasalazine (but never azathioprine). He also underwent acupuncture and was given a course of oral Comfrey root by a naturopath. He regularly received herbal tea containing Comfrey leaves as a tonic.

He developed diarrhoea and weight loss and a few weeks later, fever, abdominal pain and swelling. On examination there was ascites, tender hepatomegaly and mild peripheral oedema. He was anaemic but not jaundiced. The ascitic fluid protein content was 27 g/l. The inferior vena cava and major hepatic veins were patent at Doppler ultrasonography and venography. A liver biopsy showed hepatic veno-occlusive disease.

We think that the only possible aetiological factor was the ingestion of Comfrey which is known to contain at least 9 hepatotoxic pyrrolizidine alkaloids. This is the first case of hepatic veno-occlusive disease to result from a native British plant and is only the second published case related to Comfrey ingestion.

### EROSIVE GASTRITIS AND GASTROINTESTINAL BLEEDING IN A FEMALE RUNNER

**B. T. Cooper, S. Douglas, A. Firth and V. S. Chadwick**
**Bristol Royal Infirmary and Dunedin Hospital, New Zealand**

There are a few reports of gastric erosions with gastrointestinal bleeding in athletes but the relationship between the erosions, the bleeding and exercise has never been proved. We report a 33 year old female runner who presented with a 3 year history of dyspepsia, epigastric pain and anaemia. She related her symptoms to running. She was on no medication. Investigation revealed iron deficiency and erosive gastritis on endoscopy, but no evidence of small or large intestinal abnormality. She agreed to 3 studies designed to determine the relationship of the erosive gastritis and iron deficiency to running. In the first, it was shown endoscopically that the gastritis healed with rest, recurred on running 7–10 miles a day and healed with cimetidine despite continued exercise. Her haemoglobin and serum iron fell with exercise. The second study, using $^{51}Cr$ labelled autologous red cells, showed that there was significant faecal blood loss (>2 ml/day) during exercise with reappearance of gastritis endoscopically. The third study showed that H-2 blocker therapy prevented significant faecal blood loss during exercise (running 7–10 miles a day). We conclude that serious running can induce erosive gastritis associated with occult gastrointestinal bleeding.

### TASTE ACUITY, ZINC STATUS AND SMOKING IN CROHN'S DISEASE

**M. J. Hall and L. Downes**
**University Department of Medicine, Bristol Royal Infirmary, Bristol**

We have examined taste acuity, zinc status and smoking habits in patients with Crohn's disease and control subjects. Impairment of taste sensation and hypozincaemia are both recognised features of Crohn's disease but no correlation between taste sensitivity and plasma zinc has been documented. Cigarette smoking has also been reported to alter taste acuity.

Using a scoring system for the detection and recognition of sweet, salty, bitter and sour solutions, there was a significant difference in recognition but not detection scores between Crohn's patients (n = 45) and controls (n = 47) ($p<0.01$). Recognition, but not detection, scores correlated significantly with leukocyte zinc (n = 32, r = 0.363, $p<0.05$) in patients, but no correlation was found between taste acuity and plasma, urine or hair zinc. White cell zinc is thought to reflect tissue zinc status and was significantly lower in patients than controls ($45.0\pm2.16$ v $50.2\pm1.34$ mg/kg; mean±s.e.m.; $p<0.05$). There were significantly more smokers in the Crohn's group than among controls ($X^2 = 9.03$, $p<0.01$). In neither Crohn's nor control subjects was there a significant difference between smokers and non-smokers in taste recognition scores. We conclude that impaired taste recognition in Crohn's disease is associated with reduced leukocyte, and hence possibly tissue, zinc levels and is unlikely to be related significantly to the increased prevalence of smoking in these patients.

### SERUM ANTI-CAMPYLOBACTER PYLORIDIS ANTIBODIES—A SERODIAGNOSTIC TEST FOR GASTRITIS?

**Diane G. Newell*, Belinda J. Johnston, P. I. Reed and M. H. Ali**
***PHLS Centre for Applied Microbiology and Research, Porton Down, Salisbury, and Wexham Park Hospital, Slough, Berks**

Antibodies directed against an acid extractable antigenic preparation of *Campylobacter pyloridis* were determined, using an ELISA technique, in serum obtained from 113 patients undergoing endoscopy and from 10 non-endoscoped control patients with no history of upper gastrointestinal disease. Inflammation in antral biopsies was graded according to the criteria of Whitehead and antibody concentrations of >2.0 ug/ml were taken as a positive antibody response.

Sixty-six % (38/58) of patients with normal antral histology had negative responses whilst 92% (34/37) of patients with superficial gastritis had significantly raised antibody levels. Thirteen of 18 patients with atrophic gastritis with or without intestinal metaplasia also had raised levels. Only 2 of the 10 non-endoscoped patients had positive antibody responses. These results indicate that anti-C. *pyloridis* antibodies are significantly, but not uniquely, associated with an inflamed antral mucosa.

The raised antibody levels in those histologically normal patients may indicate a previous C. *pyloridis* infection, a non-etiologic relationship between organism and disease or a lack of specificity in the ELISA assay due to antigenic cross-reactivity with other enteric pathogens. These alternatives are under investigation.

### INCREASED PREVALENCE OF OSTEOPOROSIS IN CHRONIC LIVER DISEASE

**J. D. R. Rose, E. O. Crawley, W. D. Evans, C. Evans, H. Church, P. M. Smith and J. Compston**
**University Hospital of Wales, Cardiff**

Accurate measurements of bone mineral content in a large series of patients with chronic liver disease have not been reported. We measured bone mineral content in lumbar spine trabecular bone by quantitative computerised tomography and in radial cortical bone using single photon absorptiometry in 64 unselected patients with chronic liver disease (20 CAH, 21 alcoholic, 17 PBC and 6 others). Serum 25 hydroxyvitamin D (25 OH D) was measured by radioimmunoassay and serum parathormone (iPTH) by immunoassay. Fourteen patients (22%) had osteoporosis (a bone mineral <2 SD below the mean age-sex-matched control value). Of these, 6 had spinal osteoporosis, 10 peripheral cortical osteoporosis and 2 both. Four patients had vertebral fractures. A similar proportion of patients had osteoporosis in each group but spinal osteoporosis occurred mainly in steroid-treated CAH, whereas peripheral osteoporosis was commoner in PBC and alcoholic patients. Either elevated serum iPTH or decreased 25 OHD were found in 48% of whom one third had osteoporosis. We conclude that the prevalence of osteoporosis is increased in chronic liver disease, patients with steroid-treated CAH being particularly at risk from spinal trabecular osteoporosis. These findings suggest that risk of fracture may be increased in later years in these patients.

